# Vegetation productivity summarized by the Dynamic Habitat Indices explains broad-scale patterns of moose abundance across Russia

**DOI:** 10.1038/s41598-019-57308-8

**Published:** 2020-01-21

**Authors:** Elena Razenkova, Volker C. Radeloff, Maxim Dubinin, Eugenia V. Bragina, Andrew M. Allen, Murray K. Clayton, Anna M. Pidgeon, Leonid M. Baskin, Nicholas C. Coops, Martina L. Hobi

**Affiliations:** 1 SILVIS Lab, Department of Forest and Wildlife Ecology, University of Wisconsin-Madison, 1630 Linden Drive, Madison, WI 53706 USA; 2 NextGIS, Moscow, Russia; 3 Department of Forestry and Environmental Resources, North Carolina State University, Raleigh, NC 27607 USA; 4 Department of Wildlife, Fish and Environmental Studies, Swedish University of Agricultural Sciences, Umeå, Sweden; 5 Department of Animal Ecology and Physiology, Institute for Water and Wetland Research, Radboud University Nijmegen, Nijmegen, 6500GL The Netherlands; 6 Department of Statistics, University of Wisconsin-Madison, 1300 University Ave, Madison, WI 53706 USA; 7 Severtsov Institute of Ecology and Evolution, 33 Leninsky pr., Moscow, 117071 Russia; 8 Integrated Remote Sensing Studio, Department of Forest Resources Management, University of British Columbia, 2424 Main Mall, Vancouver, BC V6T 1Z4 Canada; 9 Swiss Federal Institute for Forest, Snow and Landscape Research WSL, Stand Dynamics and Silviculture Group, 8903 Birmensdorf, Switzerland

**Keywords:** Biogeography, Ecological modelling, Population dynamics

## Abstract

Identifying the factors that determine habitat suitability and hence patterns of wildlife abundances over broad spatial scales is important for conservation. Ecosystem productivity is a key aspect of habitat suitability, especially for large mammals. Our goals were to a) explain patterns of moose (*Alces alces*) abundance across Russia based on remotely sensed measures of vegetation productivity using Dynamic Habitat Indices (DHIs), and b) examine if patterns of moose abundance and productivity differed before and after the collapse of the Soviet Union. We evaluated the utility of the DHIs using multiple regression models predicting moose abundance by administrative regions. Univariate models of the individual DHIs had lower predictive power than all three combined. The three DHIs together with environmental variables, explained 79% of variation in moose abundance. Interestingly, the predictive power of the models was highest for the 1980s, and decreased for the two subsequent decades. We speculate that the lower predictive power of our environmental variables in the later decades may be due to increasing human influence on moose densities. Overall, we were able to explain patterns in moose abundance in Russia well, which can inform wildlife managers on the long-term patterns of habitat use of the species.

## Introduction

Human activity can cause major changes to the quality and extent of ecosystems through climate change and deforestation^1^ with major implications for biodiversity^2–4^. Predicting how wildlife responds and adapts, both in terms of occurrence and abundance, to these altered, and in some cases novel environmental conditions^5^, is important for management and conservation. Remote sensing is an effective tool to understand patterns in wildlife abundance, because imagery is acquired across broad scales and over long time periods^6^. Indeed, habitat and land cover maps based on remotely sensed data are powerful predictors of species occurrence patterns over large areas^6–8^. However, predicting abundances is more challenging, and continuous measures, such as plant productivity may be more important. That raises the question as to which remotely sensed indices best correlate with large mammal density and specifically how vegetation productivity affects herbivore densities.

The Dynamic Habitat Indices (DHIs) are remote sensing indices that summarize three measures of vegetative productivity: cumulative productivity (cumulative DHI), minimum productivity (minimum DHI), and seasonality (variation DHI)^9–13^. The DHIs can be computed from a range of satellite datasets including the Moderate Resolution Imaging Spectroradiometer (MODIS), which is aboard NASA’s Terra and Aqua satellites, developed to monitor the environmental conditions of Earth^14^. Originally, the DHIs were developed to predict species richness, and this relationship is well grounded in ecological theory^13^. For example, the species-energy hypothesis predicts that areas with high productivity are able to support a greater number of species^15–18^. Indeed, the DHIs are good predictors of bird species richness in Canada^9,10^, the US^11^, and Thailand^19^, and of amphibian, mammal, and bird species richness in China^12^, and across the globe^13^.

However, while the species-energy hypothesis focuses on species richness, energy, as represented by the DHIs, may also be useful in predicting abundance patterns within a given species’ range. In areas with higher vegetation productivity, animal home ranges are typically smaller^20–22^, and reproductive and survival rates are higher^23^. The relative importance of the DHIs to predict abundance is yet to be tested though, because abundance depends on many factors besides productivity, including availability of forage in space and time, necessary climate conditions for survival and reproduction, as well as predation and harvest pressure.

Wildlife abundance data collected across Russia provides a unique opportunity for exploring the relationship between species-abundance and vegetative productivity because of the broad spatial and temporal coverage of Russia’s wildlife surveys and Russia’s large variability in climate and vegetative productivity. Russia has monitored abundance of game species since the 1960s using winter track counts (WTC)^24,25^, aerial surveys, and hunter surveys^26^. We selected moose (*Alces alces*) for our analysis because it is a herbivore that has a large range, and is important for the subsistence economy of many parts of rural Russia and especially its indigenous people^27^. Moose densities vary considerably across Russia, and generally moose densities in European Russia, i.e., west of the Ural Mountains, are two to three times higher than those in the Asian part of Russia^28^. Moose abundance data were available at the scale of Russia’s administrative regions from 1981 to 2010. This time period is interesting because Russia has undergone radical political and economic changes since 1981, including the collapse of the Soviet Union in 1991, and the transition from a socialist government to a market economy. Especially in the early 1990s, there was reduced enforcement of environmental regulations. Rapid economic decline at this time affected human livelihoods and increased poverty^29^, as well as agricultural land abandonment^30^, and forest loss^31^. Consequently, the economic downturn affected wildlife populations primarily due to overexploitation, as people relied more heavily on wildlife for food^32,33^. However, after 2000, populations of many wildlife species rebounded, potentially due to increasing habitat availability on abandoned agricultural fields.

**Figure 1:**
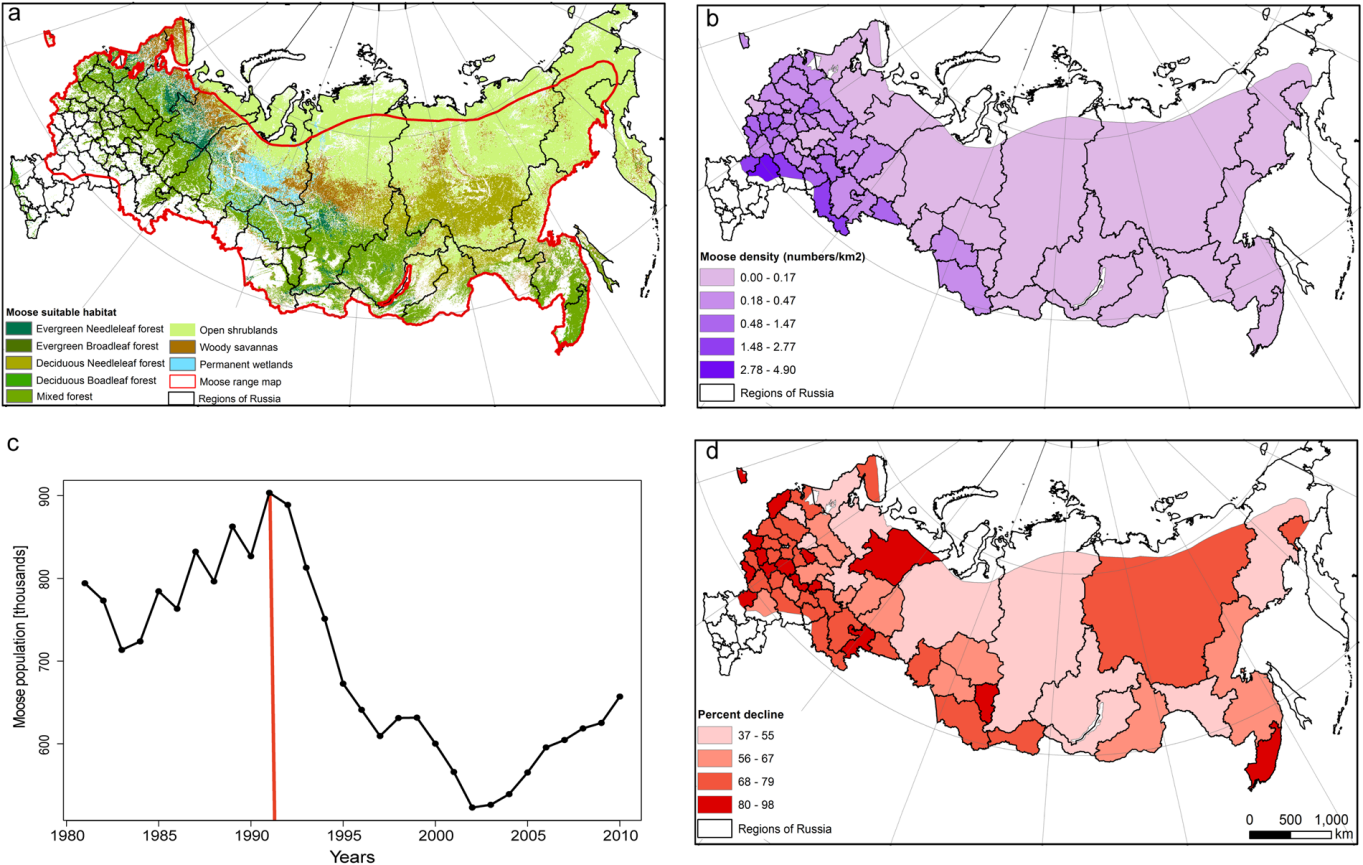
(**a**) Suitable habitat for moose based on MODIS stable land cover data from 2003 to 2012, moose range map is shown in red, (**b**) moose density (individuals per 1 km^2^) based on suitable habitat within the moose range area, (**c**) the trend of moose population for Russia from 1981 to 2010 with the red line indicating the collapse of the Soviet Union, (**d**) the decline of moose population over Russia.

The main goal of our study was to evaluate how vegetative productivity is related to moose density across Russia. Specifically, we aimed to a) explain patterns of moose (*Alces alces*) density across Russia based on remotely sensed measures of vegetation productivity (i.e., the DHIs), environmental variables (temperature and precipitation), elevation, and human influence (human footprint index, road density), and b) examine if the relationship between average moose density versus productivity and temperature differed among the last decade of Soviet time, the first decade after the collapse of the Soviet Union, and the second decade after the collapse, given the changing population trends and socioeconomic conditions during these periods (Fig. 1c). We predicted higher moose abundance in regions with higher vegetation productivity (cumulative DHI), higher minimum productivity (minimum DHI), and less variation in productivity over the course of a year (variation DHI), and that those relationships were stronger prior to the collapse of the Soviet Union than afterwards.

## Results

### Moose abundance patterns

**Figure 2:**
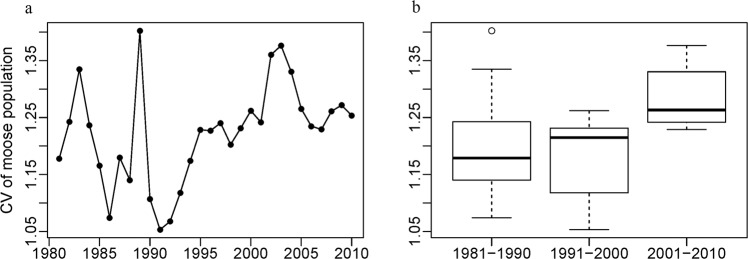
The coefficient of variation of moose population among regions by (**a**) year; and (**b**) decade.

Moose populations experienced large changes during our study period. From 1981 to 1991 moose populations grew rapidly, and reached a maximum population of approximately 900,000 moose across Russia by the end of this period (Fig. 1c). After the collapse of the Soviet Union in 1991, the moose population across Russia rapidly declined and reached a minimum in 2002 of approximately 520,000 individuals, equivalent to a decline of 42%, and in some regions the moose population declined by 98% (Fig. 1c,d). After 2002, the moose population recovered somewhat and in 2010 it reached approximately 645,000 individuals. The coefficient of variation of moose density among regions fluctuated considerably over time (Fig. 2a). Comparing the three decades, median coefficient of variation was the lowest in 1981–1990 and the highest in 2001–2010 (Fig. 2b). However, the range values of coefficient of variation for the different regions overlapped among all three decades suggesting that there was no significant change in data quality.

Based on MODIS stable land cover information we identified 10.6 million km^2^ of suitable habitat for moose within its range (Fig. 1a). In some regions, for example, Volgograd and Orenburg, there was little forest cover, resulting in a small area of suitable habitat based on the eight selected land cover classes. However, both regions are located on large rivers, and moose live in the floodplains, which were not always correctly classified in the MODIS land cover data. Thus, while the total moose population was low in these districts, densities may have been somewhat inflated because of the small habitat area in the denominator.

### DHI and moose density

**Figure 3:**
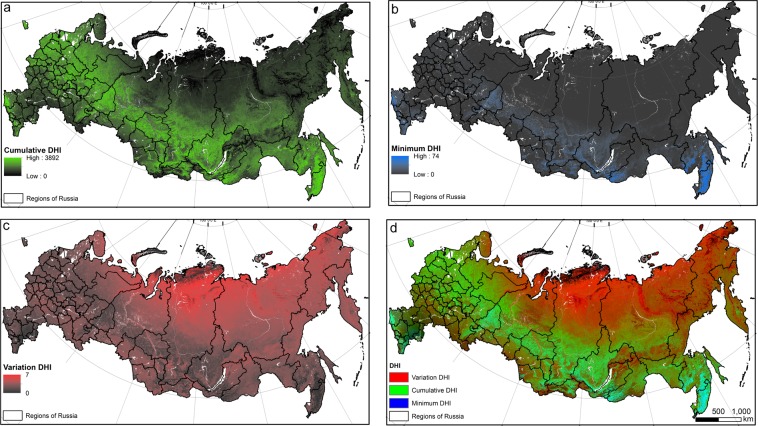
The Dynamic habitat indices based on FPAR with 8-day temporal resolution: (**a**) Cumulative Productivity - cumulative DHI, (**b**) Minimum productivity - minimum DHI, (**c**) Seasonality - variation DHI and (**d**) The three DHIs, cumulative productivity (green), minimum productivity (blue), and seasonality (red). The boundaries of regions of Russia are shown in black. Areas in white indicate no data.

The DHIs captured the temporal pattern of vegetation productivity over the Russian territory well (Fig. 3). Values for cumulative DHI were highest for mixed forests in European part of Russia and deciduous broad leaf forests in the southern part of Siberia (including the Altai, Sayans, and Sikhote-Alin mountain ranges). Minimum DHI had low values in the northern and northeastern parts of Russia, which is mainly covered by boreal forests, and high values in the southeast of the Asian part of Russia (south of Far East) and the south of Russia (Caucasus region), characterized by a mild climate. In contrast to cumulative DHI, variation DHI showed high values in the north, and especially the north-east of Russia, in tundra and taiga areas (Fig. 3).

**Figure 4:**
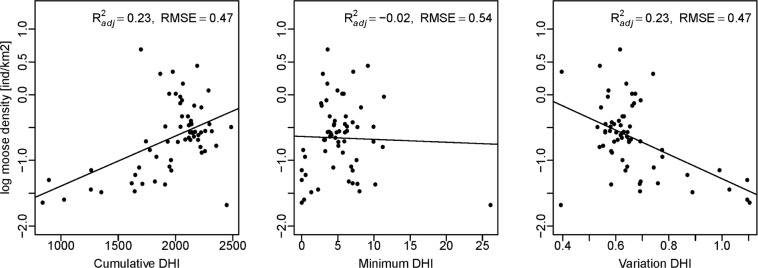
Relationship between log-transformed moose density (individuals/km^2^) from 1981 to 2010 and cumulative productivity (cumulative DHI), minimum productivity (minimum DHI), seasonality (variation DHI).

First, we evaluated each DHI individually in order to see how much moose density variation they explained. Cumulative DHI had a positive relationship with moose density (R^2^_adj_ = 0.23, P < 0.01) while variation DHI exhibited the opposite trend with low moose densities in regions with high variation DHI (R^2^_adj_ = 0.23, P < 0.01). Explained variation (R^2^_adj_) was similar for cumulative DHI and variation DHI but minimum DHI did not have a notable trend (R^2^_adj_ = −0.02, P = 0.8) (Fig. 4).

**Figure 5:**
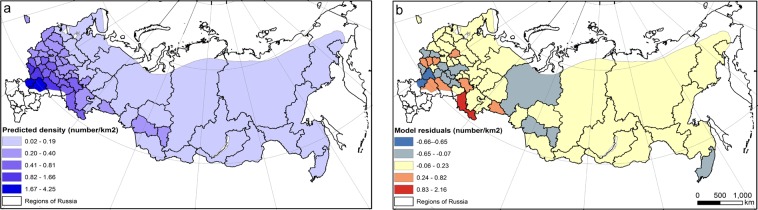
Moose density (**a**) predicted by the model, and (**b**) model residuals.

In our multiple regression models, cumulative DHI, human footprint, road density, and some BIOCLIM variables (especially annual mean temperature BIO1) were retained in the best models. Based on the best subsets selected, we examined the top three performing models and two of them included the DHIs (Table 1). The “best” model based on BIC included cumulative DHI, annual mean temperature (BIO1), temperature annual range (BIO7), and human footprint (Table 1). However, the VIF of BIO1 was 16 due to high collinearity between BIO1 and human footprint (r = 0.89), and between BIO1 and BIO7 (r = −0.85). Therefore, we refined the “best” model by removing those variables with high collinearity, and BIOCLIM variables that were clustered (see SI Figs. S1 and S2). The parsimonious model thus included cumulative DHI, annual mean temperature (BIO1), and temperature seasonality (BIO4). We calculated predicted values of moose density using the parsimonious model. Predicted moose density was most closely related to the cumulative DHI, with the highest values in the European part of Russia and a gradual decline towards the north and northeast of Russia (Fig. 5a). The highest moose densities were in the Volgograd and Rostov regions. A map of residuals of this model showed where moose densities were over- or underestimated by this model (Fig. 5b). The second-best model included only two explanatory variables: maximum temperature of warmest month (BIO5) and mean temperature of driest quarter (BIO9). To evaluate other components of the DHIs, we used a parsimonious model and fitted the models with minimum DHI and variation DHI instead of cumulative DHI. Cumulative DHI in combination with other variables performed better than variation DHI and minimum DHI, as measured by BIC, but the R^2^_adj_ was similar for all three models (Table 1).

**Table 1:** Multiple linear regressions between average moose density from 1981 to 2010 for the 62 administrative regions and our explanatory variables including the DHIs, environmental variables, and human influence variables.

Model	BIC	∆BIC	R^2^_adj_	RMSE [ind./km^2^]
Cumulative DHI + BIO1 + BIO7 + human footprint	23.66	0	0.79	0.24
Cumulative DHI + BIO1 + BIO4	24.92	1.38	0.77	0.25
BIO5 + BIO9	33.13	9.59	0.73	0.28
Variation DHI + BIO1 + BIO4	33.25	9.62	0.74	0.27
Minimum DHI + BIO1 + BIO4	36.34	12.64	0.72	0.27

We present the Bayesian Information Criteria (BIC), the adjusted coefficient of determination (R^2^_adj_), and the root mean square error (RMSE) for the top performing models (Model 1–3), and for the models that included variation DHI and minimum DHI instead of cumulative DHI.

### Differences among the three decades

**Figure 6:**
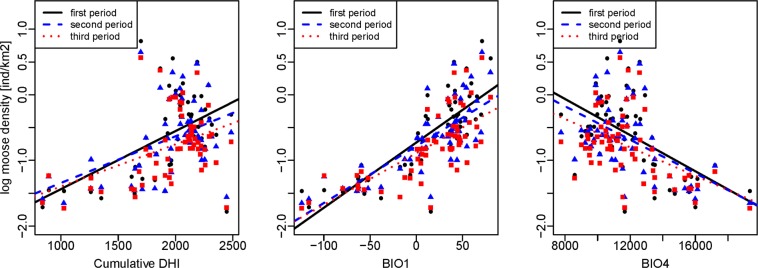
Relation between log-transformed moose density (individuals per 1 km^2^) for the three periods and cumulative DHI, annual mean temperature (BIO1), temperature seasonality (BIO4).

The most parsimonious multiple regression model for moose density in the 1980s, which we also used to evaluate moose densities for the three decades, included cumulative DHI, annual mean temperature (BIO1), and temperature seasonality (BIO4). Moose density increased with increasing values of cumulative DHI and BIO1, while it decreased for increasing values of BIO4 (Fig. 4). Interestingly, the slopes of the regression lines for the univariate models of the three decades differed only slightly (Fig. 6), but the slopes of the multivariate models differed significantly between the first and the third decade (P = 0.007), and between the second and the third decade (P = 0.013). However, there was no significant difference between the first and the second decade (P = 0.91). The relation between moose density and cumulative DHI, BIO1, and BIO4 was stronger for the first decade (R^2^_adj_ = 0.81) than the second (R^2^_adj_ = 0.73) and third decade (R^2^_adj_ = 0.67) (Table 2). Rural population was not significant in the models for any of the three decades.

**Table 2:** The most parsimonious top-ranked model for each decade in which the dependent variable was the average moose density, our unit of analysis was the administrative region (n = 62), and our explanatory variables included cumulative DHI, annual mean temperature (BIO1), and temperature seasonality (BIO4).

Period (years)	Model	R^2^_adj_	RMSE [no./km^2^]
First (1981–1990)	cumulative DHI + BIO1 + BIO4	0.81	0.25
Second (1991–2000)	cumulative DHI + BIO1 + BIO4	0.73	0.27
Third (2001–2010)	cumulative DHI + BIO1 + BIO4	0.67	0.29

We present the Bayesian Information Criteria (BIC), the adjusted coefficient of determination (R^2^_adj_), and the root mean square error (RMSE) for the models for each of the three time periods.

## Discussion

We evaluated the relationship of moose density with vegetative productivity as captured by DHIs across Russia. Our results show that univariate models based on the individual DHIs had low predictive power. However, models combining cumulative DHI with environmental variables, either with or without a proxy for human effects (e.g., human footprint), explained up to 79% of variation in moose density. Interestingly, the relationship between moose density and the DHIs and environmental variables changed significantly from the 1980s to the 2000s. The predictive power of our model based on R^2^_adj_ was highest for the 1980s and lowest for the 2000s, suggesting that other factors, that our variables did not capture, gained importance. Poaching may be one such factor, even though our proxy variables for human influence did not gain predictive power in the later decades. Another factor could be a decline in data quality after the collapse of Soviet Union, even though we did not find quantitative evidence for such a decline (Fig. 2).

Variation in moose density was best explained by vegetative productivity as captured by the cumulative DHI and temperature-related variables, and these explained 81% of variation in moose densities during the 1980s. Previous studies of ungulates have also shown that abundance of roe deer (*Capreolus capreolus*) and wild boar (*Sus scrofa*) are positively correlated with vegetative productivity^34,35^. Reproductive performance of moose is positively related to vegetative productivity, with higher twinning rates in females with good body condition^36,37^, which may be an underlying mechanism for our correlations. Interestingly, Michaud *et al*.^38^ found that the minimum levels of productivity (minimum DHI) during winter were more important for explaining abundance of moose in Canada. Minimum DHI may indicate levels of forage availability during winter, an important determinant of moose space -use during the lean winter months^39^, and forage availability during winter may also have carryover effects on calf survival the following spring thus affecting population recruitment^37,40^. However, in our analyses minimum DHI explained very little variance in moose density. This may be due to missing values arising from periods of darkness or snow cover in the northern parts of our study region^41–43^.

We predicted that higher human presence would have a negative effect on moose density. However, we did not find a strong relationship between moose density and either road density or rural populations, and the human footprint index was positively associated with moose density. Human presence typically affects wildlife negatively; for example, ungulates may alter their activity patterns in response to human disturbance^44,45^, roads improve hunter access^46^, and human development causes habitat fragmentation^47^, but human presence can also influence wildlife populations positively. Predators often avoid human-dominated areas thus providing a safe-haven for their prey^48^, humans may increase forage availability through fertilizers^49^, and logging may open forest canopies and stimulate the growth of early-successional vegetation, thereby improving habitat suitability for moose^50^. We caution though, that the positive association between moose density and the human footprint index is probably not due to a causal relationship, but rather reflect that better conditions for both people and moose are found in the same areas. Humans and moose may both preferentially select more productive areas, and human population density is often positively correlated with vegetative productivity^51^. Indeed, we found a positive relationship between human footprint and both cumulative DHI and minimum DHI, and a negative relationship with variation DHI.

In addition, rapid changes in political and economic activity can lead to changes in land use and forest cover. Immediately after the collapse of the Soviet Union, agricultural abandonment was common across Russia and especially widespread in its European part^52^. As a consequence, forest area increased^31^, potentially providing more habitat for wildlife. However, moose populations experienced high hunting pressure immediately after 1991, because of government instability and a lack of wildlife protection, resulting in an overharvesting of natural resources^32,33,53^. Political instability may have also influenced data quality because there may have been less oversight and less effort, leading to less reliable information about the status of wildlife population, and ultimately ill-advised management decisions and hunting quotas^54,55^. To reduce the effects of these potential errors, we averaged moose density over time (by decade and for the full study period). Ultimately though, there is no reason to assume that errors in the reported moose densities in a given time period were correlated with vegetation productivity, which means that low data quality would have introduced additional random variance into our models, and hence reduced their predictive power. Conversely, the high R^2^_adj_ of our models suggest that remaining errors were fairly minor. However, we caution that if there was a systematic difference in data quality among the three decades, then that would affect their relative R^2^_adj_. For example, if we assume that the data quality was highest during Soviet time, then the decrease in the predictive power of our models for each decade may be related to lower data quality, rather than being an indication that vegetation productivity was more important during the 1980s while the moose population was increasing. However, the decline in predictive power from the 1990s to the 2000s is less likely to be due to changes in data quality, because we would assume that data quality was lower during the turbulent and lawless 1990s than during the 2000s. In summary, the high predictive power of our models suggests that the available moose density data for Russia captures broad-scale patterns well, but we cannot rule out that difference in data quality among decades affected our results.

We assume that the moose population decline during the second decade was due to increasing human pressure and illegal hunting. That may be why vegetation productivity had less predictive power in models in the second and third decades (when the moose population was low). However, we caution that our proxies for human effects were not significant predictors in any of our models of moose density. Furthermore, the coefficient of variation across years of moose densities increased in the second and third decades, which may indicate a decline in data quality, but the ranges of CVs in each decade overlapped, suggesting data quality was not significantly worse in later decades. Our results for the CV across years cannot prove that data quality was consistent over time, but indicated at least that there is no significant increase in variability. If data for smaller administrative units, or even for individual transects, had been available then it would have been interested to calculate the coefficient of variation within oblast, but such data were not available to us.

In summary, the Dynamic Habitat Indices, which were originally designed to predict species richness, also provided valuable information about the productivity of ecosystems in models of animal abundance, especially when used in conjunction with bioclimatic variables. In this study, we calculated the DHIs based on the MODIS FPAR and showed that the combination of remote sensing based products incorporated in the DHIs and land cover together with climate variables are very promising for the prediction of abundances of large ungulates, such as moose. One advantage of our approach is that it is relatively robust in regards to errors in reported data, and could hence be applied to predict future moose density based on predictions of vegetation productivity. Such predictions would be even better though if they could incorporate variables that we were not able to quantify in our models, such as poaching.

## Methods

### Study area

Our study area covered most of the territory of Russia and included 69 administrative regions (13.64 million km^2^). The borders of some regions of Russia changed, and some were subdivided between 1981 and 2010. We thus analyzed 62 regions using their original borders prior to subdivision. Russia’s vast area is ideal for our research questions because it covers multiple landscape zones, and includes a diversity of topographic and vegetation types, resulting in substantial diversity of habitats and large ranges of values of the three DHIs.

Russia consists of two main parts: the East European Plain, which has little topographic relief, and the Asian section, which includes the West Siberian Plain, Central Siberian Plateau, mountain areas of Southern Siberia and the Far East where both large mountain ranges and well-drained plains occur. The dominant climate across the entire country is continental with two main seasons, winter and summer, and two transitional seasons, spring and fall. The average annual temperature is −5.5 °C, the coldest month is January (mean January temperature ranges from −38.6 °C in Yakutsk to −6.3 °C in Volgograd), and the warmest month is July (mean July temperature ranges from 19.5 °C in Yakutsk to 23.6 °C in Volgograd). Vegetation types include taiga (boreal forest), and temperate broadleaf forest. Boreal forests are dominated by pine *(Pinus sylvestris, P. sibirica)*, spruce *(Picea abies, P. obovata)*, larch *(Larix gmelinii)*, and Siberian fir *(Abies sibirica)*. Temperate broadleaf forests are dominated by birch *(Betula pendula, B. pubescens)*, aspen *(Populus tremula)*, alder *(Alnus glutinosa)*, oak *(Quercus robur)*, linden *(Tilia cordata)*, ash *(Fraxinus excelsior),* and maple *(Acer platanoides)*^56^.

### Data

#### Winter track count data, and range map

We obtained moose abundance data from the Russian Federal Agency of Game Animals for 1981–2010, based on the winter track count (WTC)^24^ for the 62 administrative regions (‘oblasts’)^26,57–61^. The WTC involves counting animal tracks that intersect fixed transects on snow, and measuring daily travel distance of surveyed species^62^. WTCs were first proposed in 1934 by A. N. Formozov, who showed how the occurrence of tracks on snow together with the length of daily travel distance are related to population density^62^. Later, his formula was refined and verified^24,25,63^. The WTC has been widely implemented in different parts of Russia starting in 1964. In 1981, the WTC became the main method for monitoring game animals (covering 14–33 species, depending on the year) in all territories of Russia that have stable snow cover. Approximately 30,500 transects were monitored in 1981 and the length of an individual transect ranged from 8 to 12 km^62^. The number of transects changed over time with fewer transects in the early 1990s (26,599 transects in 1992)^58^. We could only obtain summary abundance data at the oblast level, and did not have access to the details of transects conducted within each oblast. It is likely that the density of transects per unit area is higher in European Russia than in Siberia and the Russian Far East simply because there are far fewer people and natural resource professionals in the latter, and many areas are very remote. However, administrative regions are also much larger in the Asian part of Russia, and that counteracts a lower density of transects and ensures a sufficient number of transects to estimate wildlife population totals, and to set hunting quotas, which was the main goal of the WTC.

Moose are one of the most valuable game species in Russia and occur in almost all regions (Fig. 1). Several methods have been applied to estimate moose abundance in addition to the WTCs, including aerial surveys and hunter surveys. For this reason, the moose data are considered more reliable than those of the other species surveyed^26^. However, there are limitations of WTC data including human errors made at different stages of collection, processing, and reporting of WTC data. Moreover, data were collected over a very long period of time including a politically unstable period, and data quality may not have been consistent. To check for changes in data quality through time, we calculated the annual coefficient of variation (CV) of moose population density among regions, assuming that higher CV values indicate noisier data.

Our aim was to identify general patterns of moose density in relation to the environment, rather than disentangling the drivers of annual variation in moose density. Therefore, in the first part of our analysis, we calculated the average moose density for the entire study period over 1981–2010 for the 62 administrative regions. For the second part, we divided the study period into three decades, which captured major differences in political and socioeconomic conditions (i.e., 1981–1990, 1991–2000, and 2001–2010), and calculated the average moose density for each decade for the 62 administrative regions. For the year 1996, we had no data, and there were five missing values in other years for single regions, which is less than 0.3% of the total values. We used linear interpolation to estimate all missing values.

#### MODIS data: Dynamic habitat indices and land cover

We calculated the Dynamic Habitat Indices (DHIs) based on the Fraction of Absorbed Photosynthetically Active Radiation (FPAR) collected by the Moderate Resolution Imaging Spectroradiometer (MODIS) instrument aboard the Terra and Aqua satellite with 1-km spatial resolution and 8-day temporal resolution from 2003–2014. The DHIs capture three aspects of vegetation productivity: annual cumulative productivity (cumulative DHI), minimum productivity (minimum DHI), and seasonality (variation DHI). We calculated cumulative DHI by summing FPAR values over a year. Minimum DHI is the lowest FPAR value during a year, and variation DHI is the coefficient of variation (standard deviation divided by mean) (Fig. 3). Missing values of minimum DHI at high latitude due to winter darkness were set to zero. Although annual data of DHI are available (since 2003), our analyses focus on average moose densities between 1981 and 2010 and hence the general patterns of vegetative productivity. Therefore, we estimated the composite DHIs, which are calculated from the median FPAR values for each 16-day period from 2003 to 2014^11,13^.

To calculate moose density, we estimated the amount of suitable habitat within the moose range of each region. We used the range map for moose from the same source as WTC data (“The game animal’s analytical materials”, Lomanov *et al*. 1996) to calculate the area of the region that was within the range of moose. To assess suitable habitat within the moose range, we used a map of stable land cover, which we derived from the MODIS land cover product with 500-m resolution for 2003–2012^64^. If one land cover type remained stable for more than half of the years from 2003–2012 for a given pixel, we defined it as stable cover, otherwise, we did not include that pixel. Based on this stable land cover map we defined the following classes as suitable habitat for moose: 1-evergreen needle leaf forest, 2-evergreen broadleaf forest, 3-deciduous needle leaf forest, 4-deciduous broadleaf forest, 5-mixed forest, 7-open shrub lands, 8-woody savannas, and 11-permanent wetland (Fig. 1a)^65,66^. We projected our data to an Albers equal area conic projection (Datum D European 1950) to calculate the suitable habitat area for each individual region.

#### Environmental variables and elevation

**Figure 7:**
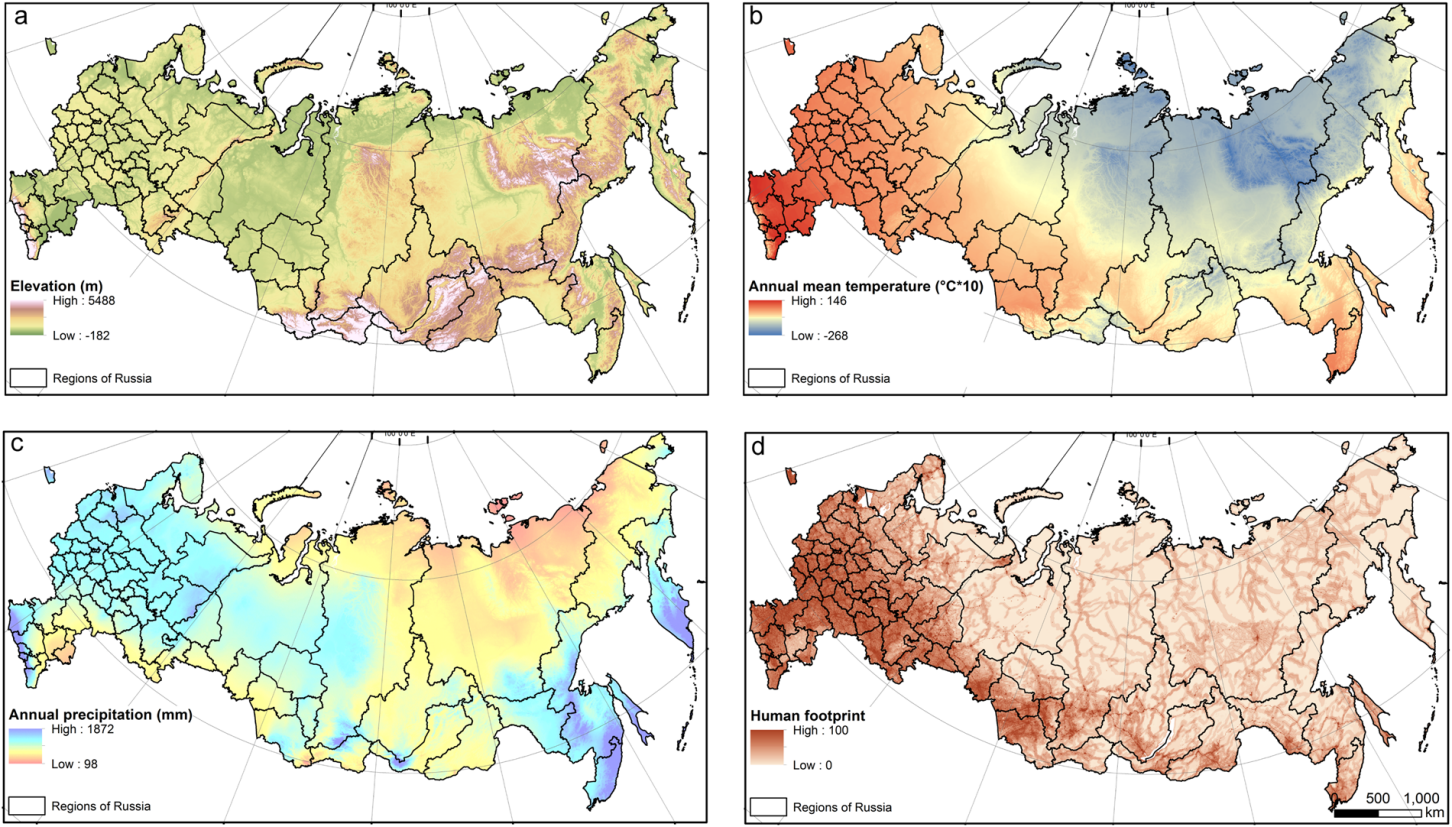
Spatial patterns of some of our explanatory variables: (**a**) elevation (m), (**b**) annual mean temperature (BIO1, °C*10), (**c**) annual precipitation (BIO12, mm), (**d**) human footprint.

To capture climate and environmental conditions in addition to the DHIs, we obtained nineteen BIOCLIM variables from 1950–2000 period^67^ (Table 3) and elevation data with 1-km resolution from WorldClim (http://worldclim.com). The elevation data in WorldClim is based on the Shuttle Radar Topography Mission (SRTM). We calculated mean values for all variables within suitable moose habitat for each of the 62 regions (Fig. 7).

**Table 3:** Environmental variables from WorldClim.

Variables	Description
BIO1	annual mean temperature (°C),
BIO2	mean diurnal range (mean of monthly(max-min) (°C))
BIO3	isothermally (mean diurnal range/temperature annual range)
BIO4	temperature seasonality (standard deviation*100)
BIO5	maximum temperature of the warmest month (°C)
BIO6	minimum temperature of the coldest month (°C)
BIO7	temperature annual range (maximum temperature of warmest month minimum temperature of coldest month (°C))
BIO8	mean temperature of wettest quarter (°C)
BIO9	mean temperature of driest quarter (°C)
BIO10	mean temperature of warmest quarter (°C)
BIO11	mean temperature of coldest quarter (°C),
BIO12	annual precipitation (mm)
BIO13	precipitation of wettest month (mm)
BIO14	precipitation of driest months (mm)
BIO15	precipitation seasonality (coefficient of variation)
BIO16	precipitation of wettest quarter (mm)
BIO17	precipitation of driest quarter (mm),
BIO18	precipitation of warmest quarter (mm)
BIO19	precipitation of coldest quarter (mm)

#### Human influence

We used three metrics to investigate human influences on moose populations. The first measure was the Human Footprint Index (http://sedac.ciesin.columbia.edu/data/set/wildareas-v2-human-footprint-geographic), available at 1-km resolution, which integrates human population pressures (population density), human land use and infrastructure (built-up areas, nighttime lights, land use/land cover), and human access (coastlines, roads, railroads, navigable rivers)^68^ (Fig. 3).

The second measure of human effects was road data for the Russian Federation, available from DIVA-GIS (http://www.diva-gis.org) from 1992. We projected the road data to an Albers equal area conic projection to calculate the length of roads. The road density of a region was calculated as the length of roads within the region divided by its area.

The third measure of human effects was rural human population data, available from the Russian Federal Service of State Statistics for 1991–2010. Rural populations include all those situated outside of cities^69^. We calculated average rural population density for two of the decades (1991–2000, 2001–2010, no data were available for 1981–1991).

### Statistical analysis

#### Models predicting spatial patterns in moose abundance

For the statistical analysis of WTC data, we parameterized multiple linear regression models. We calculated the average moose density between 1981 and 2010 for each of the 62 administrative regions. We estimated moose density by dividing the WTC total population estimates by the area of suitable moose habitat within the range of moose in each region (Fig. 1b). The dependent variable in all of our regression models was average moose density (a) for the entire study period, (b) per decade, which we log-transformed to normalize the data. Based on residual plots there were no outliers. Explanatory variables in the multiple regression included the DHIs, the BIOCLIM variables (11 temperature variables, 8 precipitation variables), elevation, road density, Human Footprint Index, and rural population. We calculated Pearson’s univariate correlation coefficients among all pairs of explanatory variables to check for potential multicollinearity (SI Fig. S1), conducted a hierarchical cluster analysis of the bioclimatic variables based on squared Spearman correlation (SI Fig. S2), and excluded those that were highly correlated.

**Figure 8:**
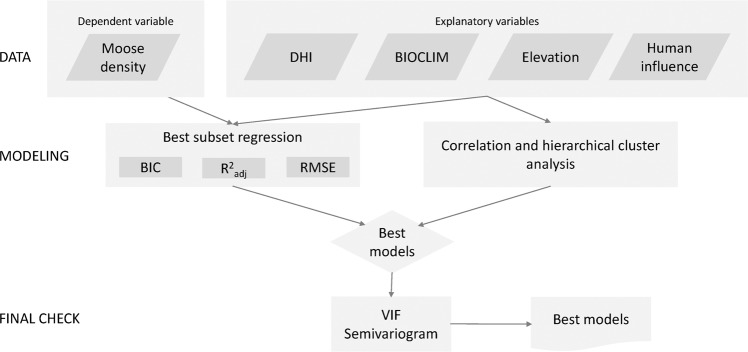
Workflow of the statistical analysis and model selection.

We applied best subset regression, which fits all possible models and identifies a set of good models^70^. To identify the most parsimonious model, we used the Bayesian Information Criteria (BIC), which applies a larger penalty for additional variables, to rank competing models^71^, the adjusted coefficient of determination (R^2^_adj_) to estimate how much of the variation in the response variable was explained by the model, and the root mean square error (RMSE) to estimate the predictive accuracy of the model. After selecting several good models with the lowest BIC, we assessed multicollinearity of the selected explanatory variables by examining the variance inflation factor (VIF) for each variable, applying a threshold of VIF < 10^72^. We calculated longitude and latitude of centroids, in degrees, for each region. Finally, we used semi-variograms to check for spatial autocorrelation in model residuals, and did not find any significant autocorrelation (results not shown, see workflow of the statistical analysis Fig. 8). We used the most parsimonious model to predict moose density over the moose range and map residuals of our model.

#### Models for the three decades

In addition to the long-term average moose densities from 1981 to 2010, we also parameterized models for average moose density for each decade (1980s, 1990s, and 2000s) for the 62 administrative regions. The first period (1981 to 1990) captures the decade before the collapse of the Soviet Union, the second period (1991 to 2000) includes the transition from a socialist government to a market economy, and the third period (2001 to 2010) was after the initial transition period. We selected the most parsimonious model from the first part of the analysis and refitted the model for each decade. We compared the intercepts and slopes across the decades using an additional sums of squares test. The best model that we selected from the first part of the analysis did not include any proxy variable for human effects. However, humans may have affected moose abundances more after the collapse of the Soviet Union, which is why we included the variable rural population in the models for the second and the third decades.

We performed our analyses in R version 3.3.1^73^, using the following R packages: Hmisc^74^ to run cluster analysis, leaps^75^ to perform best model selection, geoR^76^ for semivariograms.

## Supplementary information

Supplementary Information.

## Data Availability

All datasets and R code are available in the SI accompanying the manuscript. The DHIs can be downloaded at http://silvis.forest.wisc.edu/data/dhis/.
