# Supplementary material for: Vegetation productivity summarized by the Dynamic Habitat Indices explains broad-scale patterns of moose abundance across Russia
Source: Sci Rep. 2020 Jan 21;10:836. doi: 10.1038/s41598-019-57308-8 (PMC6972780; doi:10.1038/s41598-019-57308-8)
Supplement: Supplementary file 1 — Supplementary Information. [file 41598_2019_57308_MOESM1_ESM.pdf]

Supplementary Information

Vegetation productivity summarized by the Dynamic Habitat Indices explains broad-scale patterns of moose abundance across Russia

Elena Razenkova, Volker C. Radeloff, Maxim Dubinin, Eugenia V. Bragina, Andrew M. Allen, Murray K. Clayton, Anna M. Pidgeon, Leonid M. Baskin, Nicholas C. Coops, Martina L. Hobi

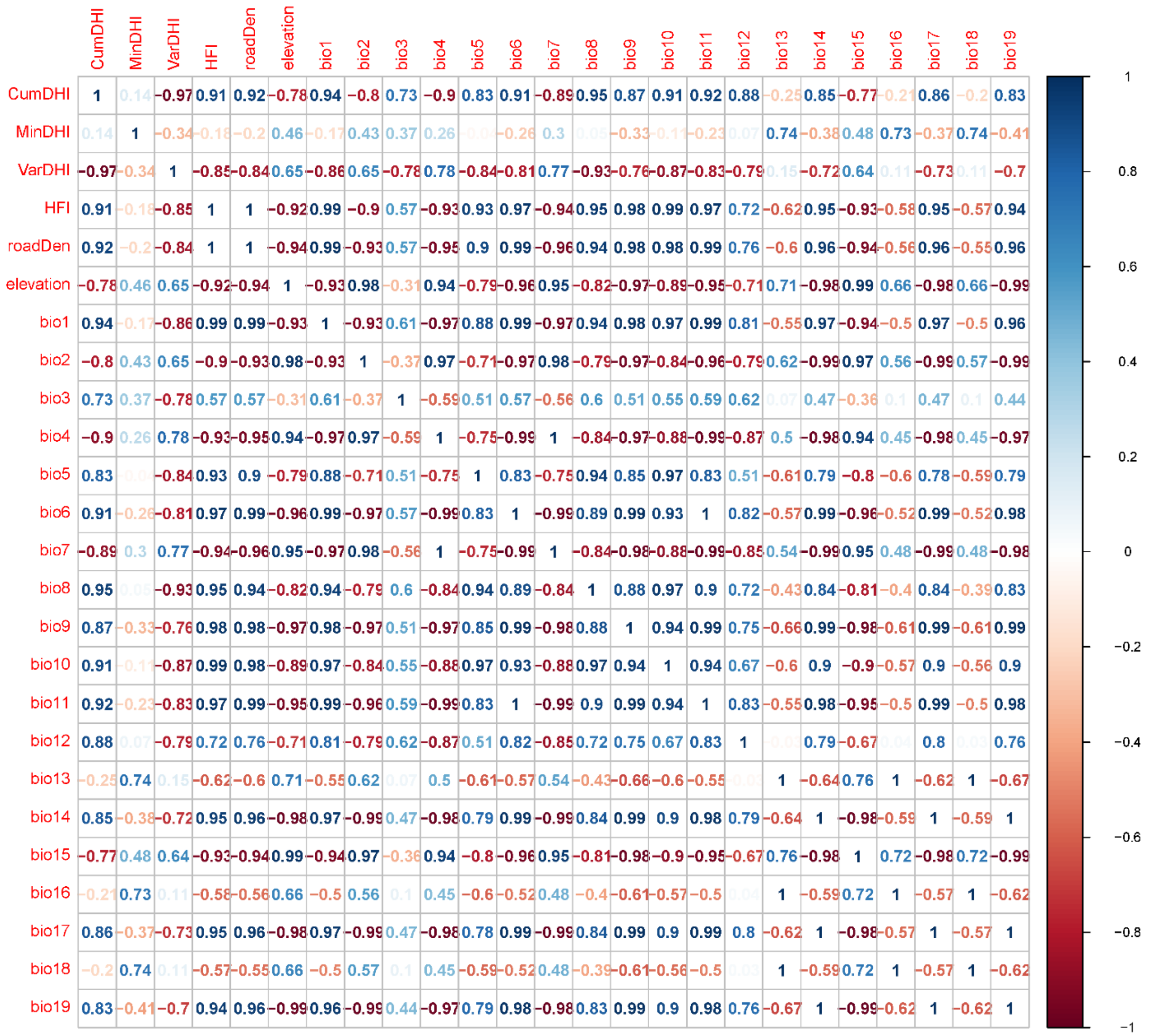

Figure S1: Pearson's correlation for explanatory variables where CumDHI is cumulative DHI, MinDHI – minimum DHI, VarDHI – variation DHI, HFI - human footprint index, roadDen – road density.

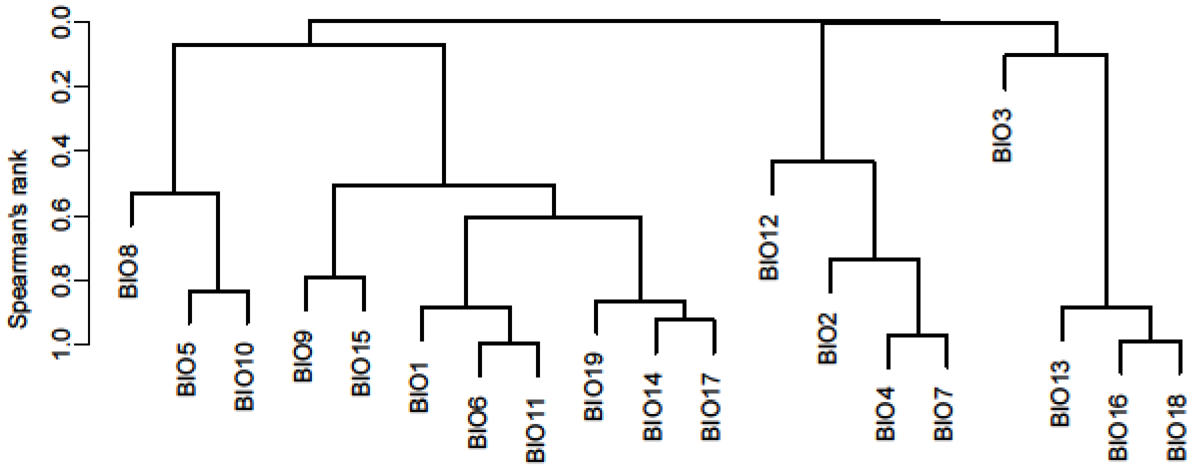

Figure S2: A hierarchical cluster analysis for the environmental variables from WorldClim using the squared Spearman's rank correlation coefficient.

```
##########################################################################
# R code for Elena Razenkova, Volker C. Radeloff, Maxim Dubinin, Eugenia V. Bragina,
#Andrew M. Allen, Murray K. Clayton, Anna M. Pidgeon, Leonid M. Baskin,
# Nicholas C. Coops, Martina L. Hobi "Vegetation productivity summarized by the
# Dynamic Habitat Indices explains broad-scale patterns of moose abundance across Russia",
##########################################################################

requiredPackages <- c('corrplot', 'car', 'leaps',
                      'psych', 'Hmisc') #The packages I need

# install/load required packages:
if (exists("requiredPackages")) {
  # install required packages that are not installed yet:
  new.packages <- requiredPackages[!(requiredPackages %in%
                                       installed.packages()[,"Package"])]
  if(length(new.packages)) {
    install.packages(new.packages)
  }
  # load required packages:
  lapply(requiredPackages, library, character.only=T)
}
#########################
###Moose abundance data##
#########################
working_dir ="C:/Users/Documents" #set your working directory
setwd(working_dir)
moose_total=read.csv("moose_interpolated.csv")
```

```
##########################
###Population dynamics###
##########################
moose_clean=moose_total[,-1] #remove years from table
moose_sum_by_year=apply(moose_clean, 1, sum, na.rm=TRUE) #calculating sum values for each year
###Figure 1c
plot(moose_total$year,moose_sum_by_year, type= "o", col= "black", cex.main =2, lwd= 2.5,
    xlab="Years", cex.axis=1.5, cex.lab=1.8,ylab="Population, thousands")
###########################
### Data quality check###
##########################
#for 1981-1990
cv1=apply (moose_clean[1:10,], 1, function (moose_total)(sd(moose_total, na.rm=T)/mean(moose_total, na.rm=T)))
years1=moose_total[1:10,1]
#for 1991-2000
cv2=apply (moose_clean[11:20,], 1, function (moose_total)(sd(moose_total, na.rm=T)/mean(moose_total, na.rm=T)))
years2=moose_total[11:20,1]
#for 2001-2010
cv3=apply (moose_clean[21:30,], 1, function (moose_total)(sd(moose_total, na.rm=T)/mean(moose_total, na.rm=T)))
years3=moose_total[21:30,1]
#Figure 2a
CV_by_year=apply (moose_clean, 1, function (moose_clean)(sd(moose_clean, na.rm=T)/mean(moose_clean, na.rm=T)))
plot(CV_by_year~moose_total$year, ylab = "CV of moose population", pch=16,
    lwd= 1.2, xlab=" ", cex.axis=1.2, cex.lab=1.2,type= "o")
```

```
#Figure 2b
boxplot(cv1, cv2, cv3, main="MOOSE", ylab="CV", names=c("1981-1990","1991-2000","2001-2010"))
#########################
#Table with all explanatory variables
##########################
data = read.csv("moose_explanatory_variables.csv", header=TRUE)
logMooseDensity=log10((data$mooseMean*1000)/(data$AREA*data$habitat))
roadDen=(data$roadLength)/(data$AREA)
data_extension=cbind(data,logMooseDensity,roadDen)
##########################
#Correlation matrix
#########################
#Figure 1S
res=cor(data_extension[c(9:11,8,38,12:31)])
corrplot(res, type = "upper", order = "hclust")
corrplot(res,  order = "hclust")
library(corrplot)
C1 <- rcorr(res, type = "pearson")
corrplot(C1$r, method = "number")
##########################
#Cluster analysis
#########################
#Figure 2S
plot(varclus(~., data=data[,13:31]), las=1, cex.lab=1.5)
##########################
#Best subset model selection
#########################
```

```
leaps=regsubsets(logMooseDensity~CumDHI+MinDHI+VarDHI+roadDen+HFI+elevation+

bio1+bio2+bio3+bio4+bio5+bio6+bio7+bio8+bio9+bio10+bio11+bio12+bio13+bio14+bio15+
            bio16+bio17+bio18+bio19, intercept=T, nbest=1, nvmax=12,data=data_extension)
par (mfrow=c(1,1))
colfunc=colorRampPalette(c("dark blue", "light blue"))
plot(leaps,scale=c("bic"),col=colfunc(10), main="Moose")
#Best models
mod_best=lm(logMooseDensity~CumDHI+bio1+bio4, data=data_extension)
layout(matrix(c(1,2,3,4),2,2)) # optional 4 graphs/page
plot(mod_best)
#Table 2
results=summary(mod_best)
outlierTest(mod_best)# Bonferroni p-value
(data.vif=vif(mod_best)) #VIF
(RMSE=round(sqrt(mean(residuals(mod_best)^2)), digits=5))  #RMSE
(round(BIC(mod_best), digits=2))    #BIC
###########################
#Semivariogram
##########################
library(geoR)
resud=residuals(mod_best)
data_extension1=cbind(data_extension, results$residuals)
datvar=as.data.frame(cbind(results$residuals, data_extension1$centx, data_extension1$centy))
gengeodat=as.geodata(datvar, header=F, coord.col=2:3, data.col = 1)
plot(gengeodat)
genvario2 <- variog(gengeodat, max.dist = 65,  uvec = 20)
plot(genvario2 , pch=16, xlab="distance [degrees]", main="Semivariance for moose")
```

```
##########################
#Three periods
#########################
logMooseDensity1=log10((data$mooseMean1*1000)/(data$AREA*data$habitat)) #for 1981-1990
logMooseDensity2=log10((data$mooseMean2*1000)/(data$AREA*data$habitat))#for 1991-2000
logMooseDensity3=log10((data$mooseMean3*1000)/(data$AREA*data$habitat)) #for 2001-2010
mod_best1=lm(logMooseDensity1~CumDHI+bio1+bio4, data=data_extension)
mod_best2=lm(logMooseDensity2~CumDHI+bio1+bio4, data=data_extension)
mod_best3=lm(logMooseDensity3~CumDHI+bio1+bio4, data=data_extension)
#Table 3
summary(mod_best1)
outlierTest(mod_best1)# Bonferroni p-value
(data.vif=vif(mod_best1)) #VIF
(RMSE=round(sqrt(mean(residuals(mod_best1)^2)), digits=5))  #RMSE
(round(BIC(mod_best1), digits=2))    #BIC
```
